# Category-ComBat with distribution coefficient: a scale-adaptive harmonization algorithm for multicenter radiomic feature standardization

**DOI:** 10.3389/fonc.2026.1896994

**Published:** 2026-09-14

**Authors:** Xiaolei Zhang, Xiaona Wang, Wei Wang, Lijun Lu, Mohammad Hamiruce Marhaban, Xianling Dong, Yanli Liu

**Affiliations:** 1 Department of Biomedical Engineering, Chengde Medical University, Chengde, China; 2 Department of Radiology, Dalian Women and Children’s Medical Group, Dalian, China; 3 The Affiliated Hospital of Chengde Medical University, Chengde, China; 4 School of Biomedical Engineering and Guangdong Provincial Key Laboratory of Medical Image Processing, Southern Medical University, Guangzhou, China; 5 Faculty of Engineering, Universiti Putra Malaysia, Serdang, Malaysia

**Keywords:** algorithm, category-ComBat, discriminative, multicenter harmonization, radiomics

## Abstract

**Introduction:**

Multicenter radiomic studies are fundamentally constrained by inter-center feature variability arising from differences in scanner hardware, acquisition protocols, and reconstruction parameters. Existing harmonization methods, including ComBat, treat these sources of variability as undifferentiated batch noise and primarily correct feature distribution location, leaving scale-level heterogeneity unaddressed. This study proposes Category-ComBat with Distribution Coefficient (Category-ComBat-ω), a two-level harmonization framework for multicenter CT radiomics.

**Methods:**

A total of 1,789 pulmonary nodule CT images were retrospectively collected from three public datasets (LIDC-IDRI, NSCLC-Radiomics, and Lung-PET-CT-Dx), with 93 radiomic features extracted per case. Category-ComBat reconstructs the ComBat framework by explicitly separating imaging-related variability into systematic components, including scanner type, acquisition protocol, and reconstruction kernel, and random components. Individualized least-squares estimation was used to estimate systematic biases, together with category-stratified mean normalization. A distribution coefficient ω ∈ (0, 1] was introduced as an orthogonal scale-adjustment mechanism to adaptively compress feature distribution spread without perturbing category-level means. Performance was evaluated using Wilcoxon tests, Q-Q plots, three-dimensional principal component analysis (PCA), probability density plots, coefficient of variation (COV), intraclass correlation coefficient (ICC), and downstream machine learning classification experiments.

**Results:**

Category-ComBat effectively removed systematic inter-center variability while preserving benign-malignant discriminative separation. Introduction of ω progressively improved feature reproducibility and inter-center consistency as ω decreased. The best overall performance, evaluated using ICC, COV, and classification AUC, was observed for ω values between 0.5 and 0.9. Scale compression remained independent of location adjustment, confirming the orthogonality of the two-level design. Category-ComBat achieved the highest classification performance, with the mean AUC increasing from 0.84 for the Original features and 0.82 for ComBat to 0.89 after Category-ComBat harmonization.

**Discussion:**

Category-ComBat-ω provides a principled approach to multicenter radiomic harmonization by addressing both systematic imaging-related confounding and scale heterogeneity. Its tunable distribution coefficient enables flexible adjustment of feature distribution spread while preserving category-level means, providing an adaptable framework for different multicenter radiomic study requirements.

## Highlights

- Category-ComBat decomposes imaging factors into systematic and random components for precise multicenter radiomic harmonization.
- Removal of the systematic factor component $X_{r}\hat{\beta_{r}}$ improves distributional conformity and empirical Bayesian estimation accuracy.
- A tunable distribution coefficient ω0.5 ≤ω≤0.9 balances feature consistency with biological information preservation.
- Downstream machine learning validation demonstrates preserved or improved classification performance after harmonization.

## 1 Introduction

Radiomics refers to the high-throughput extraction of quantitative imaging features from medical images—including morphological, intensity-based, and textural descriptors—and their systematic association with clinical endpoints such as pathological diagnosis, treatment response, and prognosis (1, 2). By converting routine radiological images into mineable data, radiomics offers the potential to augment clinical decision-making without additional invasive procedures (3, 4). Over the past decade, radiomic approaches have been applied across a wide spectrum of oncological contexts, including lung, breast, prostate, and hepatocellular carcinomas, demonstrating consistent associations with histopathological subtypes, genomic signatures, and survival outcomes (5–7).

The translational promise of radiomics, however, is fundamentally constrained by the sensitivity of radiomic features to acquisition and reconstruction conditions. Features extracted from CT images are known to vary substantially across scanner manufacturers, tube voltage and current settings, slice thickness, field of view, and reconstruction kernels (8, 9). When data are aggregated from multiple institutions—as is necessary for adequately powered multicenter studies—these technical sources of variability introduce systematic inter-center bias that is confounded with the biological signal of interest (10, 11). Without harmonization, cross-center radiomic models suffer from poor generalizability, inflated false discovery rates, and irreproducible findings (12, 13).

To address inter-center variability, several harmonization strategies have been proposed. Image-level standardization approaches, such as histogram normalization and intensity standardization, attempt to reduce acquisition-related differences prior to feature extraction (14). Feature-level methods operate downstream of extraction, applying statistical correction directly to the feature matrix. Among these, the ComBat algorithm—originally developed for genomic batch correction (15)—has been most widely adopted in radiomic harmonization (16, 17). ComBat employs an empirical Bayes framework to estimate and remove additive (location) and multiplicative (scale) batch effects under the assumption that batch effects are uniform across all observations within a center. Its application to radiomic features has demonstrated meaningful improvements in inter-scanner reproducibility as measured by Intraclass Correlation Coefficients (ICC) and Coefficient of Variation (COV) (11, 18).

Nevertheless, standard ComBat presents several limitations when applied to radiomic data. First, it treats the entire imaging center as a homogeneous batch, conflating qualitatively distinct sources of variability: systematic factors that are deterministically linked to scanner configuration and acquisition protocol, and random factors attributable to operator behavior, patient positioning, and physiological motion (19). This undifferentiated treatment prevents targeted correction of the dominant systematic components, risks over-correction of biologically meaningful variance, and may reduce the discriminative power of harmonized features for downstream classification or regression tasks (20). Second, standard ComBat corrects only the location of feature distributions—adjusting center-specific additive offsets—while leaving the scale structure of the harmonized distribution unchanged except through a global multiplicative term that is not sensitive to category-level distributional differences (21, 22).

These limitations motivate the development of Category-ComBat, an enhanced harmonization framework that restructures the ComBat model to explicitly separate systematic imaging-related variability from random variability within each center (23). By decomposing the batch effect into identifiable systematic components—scanner manufacturer, acquisition protocol, reconstruction kernel—and estimating their contributions via individualized least-squares within each imaging category, Category-ComBat achieves more precise removal of technically induced bias while preserving the category-level distributional structure required for classification tasks such as benign-malignant discrimination of pulmonary nodules. Mechanistically, the algorithm incorporates a systematic factor matrix and its corresponding regression vector into the data standardization process, enabling the model to explicitly fit and remove variations attributable to deterministic imaging-system factors. Separately, additive and multiplicative error terms are defined to capture and reduce the residual variability introduced by random factors such as operator behavior, patient positioning, and physiological motion. Validation against standard ComBat using Wilcoxon signed-rank tests, Q-Q plot analysis, and three-dimensional PCA demonstrated superior distributional alignment and better preservation of discriminative feature separation (24).

However, even after location-based harmonization by Category-ComBat, residual scale heterogeneity may persist across centers, reflecting differences in the dispersion of feature distributions that are not fully captured by additive or multiplicative correction of the mean (25). Feature distribution spread—as quantified by COV and the tails of inter-center density plots—has been shown to influence the stability and reproducibility of radiomic signatures independently of mean-level alignment (19, 26). This residual scale variability represents an orthogonal dimension of harmonization that the base Category-ComBat algorithm does not explicitly address.

To resolve this limitation, the present study introduces a distribution coefficient ω as a tunable scale-adjustment mechanism appended to the Category-ComBat framework, yielding the integrated algorithm Category-ComBat with Distribution Coefficient (Category-ComBat-ω). The coefficient ω ∈ (0, 1] modulates the spread of harmonized feature distributions in a continuous and category-preserving manner: values approaching 0 compress the distribution toward the category mean, progressively reducing inter-subject dispersion and improving reproducibility metrics, while ω = 1 recovers the unscaled Category-ComBat output. Critically, ω operates orthogonally to the location adjustment performed by Category-ComBat, ensuring that category-level mean normalization and scale compression are independently controllable.

The primary objectives of this study are: (1) to present the unified theoretical formulation of Category-ComBat-ω; (2) to empirically validate the algorithm on a multicenter pulmonary nodule CT dataset; (3) to characterize the effect of ω on feature reproducibility (COV), inter-center consistency (ICC), and distributional alignment across the full range ω ∈ (0, 1]; and (4) to provide practical guidance on ω selection for harmonization pipelines with different reproducibility-information tradeoff requirements. The algorithm is implemented as an open-source module compatible with standard radiomic workflows.

## 2 Materials and methods

### 2.1 Algorithm design

#### 2.1.1 Traditional ComBat model

The ComBat algorithm assuming that radiomics feature data includes data from centers i∈1,2,…,m, sample j∈1,2,…,N and feature f∈1,2,…,F, The ComBat model is modeled as follows (Equation 1):

(1) $$Y_{ijf}=\alpha_{f}+X\beta_{f}\;+\gamma_{if}+\delta_{if}\epsilon_{ijf}$$

Where, *Y_ijf_* represents the value of the radiomics feature, *α_f_* is the overall measurement value of the feature, X is the design matrix of the radiomics feature samples, and *β_f_* is the regression coefficient of the vector X. The *ϵ_ijf_* error term can be assumed to follow a standard normal distribution with an expected value of zero and a variance of σ^2^. γ*_if_* and *δ_if_* are the additive and multiplicative error terms of the multicenter effect, respectively.

Although ComBat has been widely applied to radiomic feature harmonization, it was designed for genetic data and carries fundamental limitations when applied to imaging studies. The covariate matrix X does not distinguish between biologically relevant covariates and imaging-system–related systematic factors, such as scanner type, acquisition parameters, and reconstruction protocols. Consequently, ComBat cannot separately model systematic imaging variability and random measurement noise, resulting in incomplete harmonization and potential over-correction of biologically meaningful variance.

#### 2.1.2 Category-ComBat model

To overcome these limitations, we propose the Category-ComBat algorithm, which reconstructs the ComBat framework by (1) introducing category-level mean normalization to preserve biological variability across diagnostic classes, and (2) explicitly separating systematic imaging factors from random factors. As summarized in **Table 1**, factors influencing radiomic features are classified into two types:

**Table 1:** Factors in radiomics features and their classifications.

Factor category	Factor name	
Random factor	Electromagnetic interference, vibration, Air cleanliness environment Autocorrelation, Agency site, operator, Power fluctuations, Patient motion, motion artifacts	
Systematic factor	Difference in equipment	Different detector systems, scanner type, Coil Sensitivity, Positional and Physiological, Changes During, Acquisition, Magnetic field changes in MRI
	Differences in protocol	Voltage, Tube current, Exposure time, Slice thickness, Voxel size, Reconstruction kernel, Reconstruction protocol, Field of view, Detector Configuration

Systematic factors: deterministic, reproducible sources of variability arising from imaging equipment and acquisition protocols, including scanner type, detector configuration, tube voltage, tube current, slice thickness, voxel size, reconstruction kernel, and convolution protocol.

Random factors: stochastic, unpredictable sources of variability including electromagnetic interference, operator performance, patient motion, motion artifacts, vibration, and environmental conditions.

As shown in **Figure 1**, The model comprises three components:

**Figure 1:**
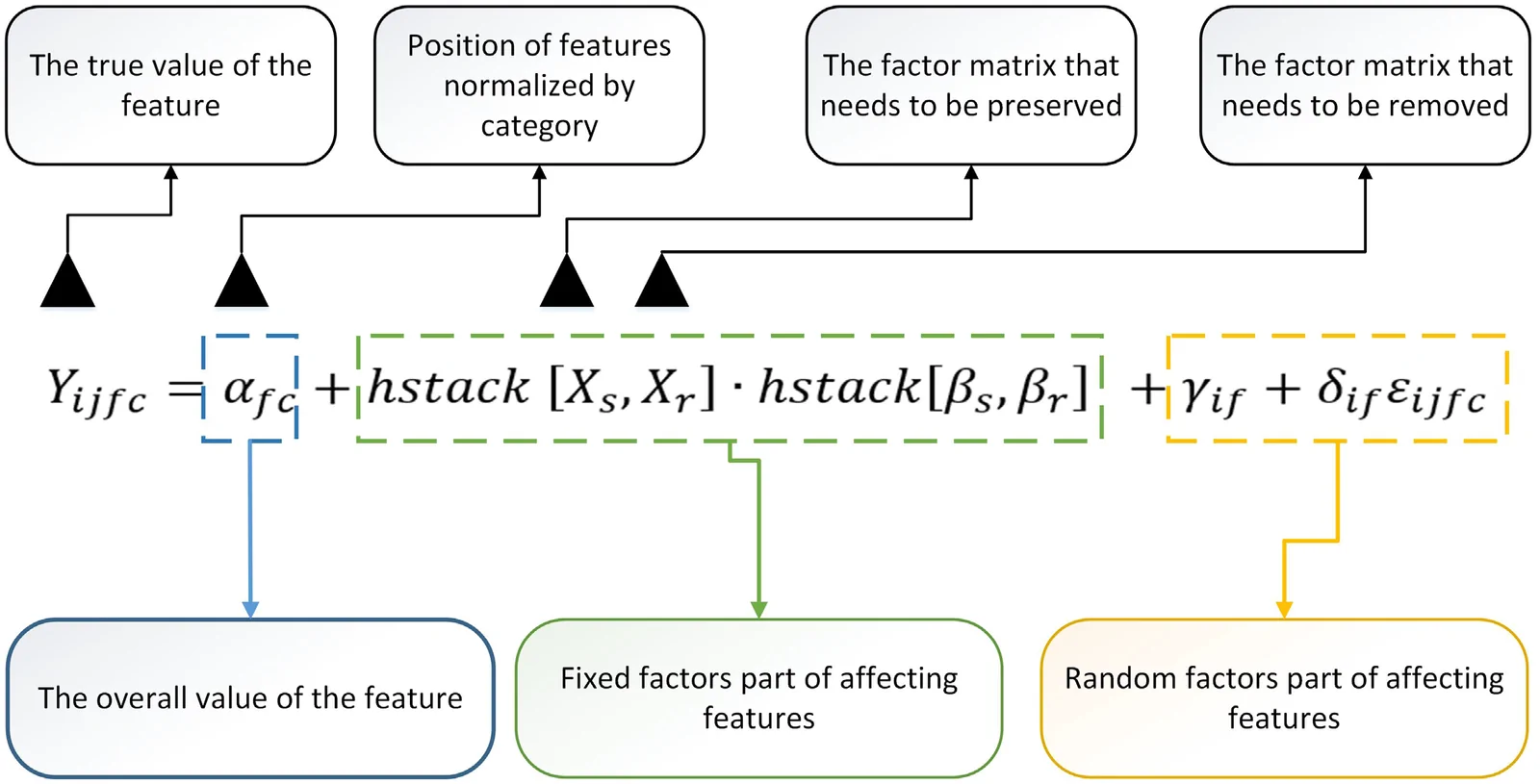
The design rationale of the Category-ComBat algorithm model.

Component 1 — Category-level mean (*α_fc_*): Rather than estimating a single global mean *α_f_* as in ComBat, Category-ComBat estimates a category-specific mean *α_fc_* for each diagnostic class *c* (e.g., benign vs. malignant). This preserves the biological distributional differences between classes throughout harmonization.

Component 2 — Systematic factor correction (*X_r_*, *X_s_*, *β_r_*, *β_s_*): *X_r_* is the systematic factor design matrix encoding per-case imaging acquisition conditions (manufacturer, scanner model, slice thickness, tube current, tube voltage, exposure time, and convolution kernel), with *β_r_* as its individualized least-squares regression vector. *X_s_* is the biological covariate preservation matrix with regression vector *β_s_*. The operator hstack[·] denotes horizontal matrix concatenation, enabling *X_s_β_s_* and *X_r_β_r_* to be handled independently without introducing confounding between biological and technical effects.

Component 3 — Random factor correction (*γ_if_*, *δ_if_*): The additive error term *γ_if_* and multiplicative error term *δ_if_* jointly model the residual stochastic variability *ϵ_ijfc_* attributable to random factors. It has been established that this additive–multiplicative error structure effectively captures random-factor–induced variation across diverse imaging settings.

#### 2.1.3 Category-ComBat parameter estimation

The parameters ${\hat{\alpha }}_{fc}$, ${\hat{\beta }}_{f}$ and ${\hat{\sigma }}_{f}$ were estimated using moment estimation and least squares. Normalized sample data *Z_ijfc_* can be calculated by (Equation 2):

(2) $$Z_{ijfc}=\frac{\left(Y_{ijfc}-{\hat{\alpha }}_{fc}-X{\hat{\beta }}_{f}\right)}{{\hat{\sigma }}_{f}}$$

The standardized data *Z_ijfc_* conforms to a normal distribution of $\text{N }\left(\varphi ,\delta^{2}\right)$. The parameter *φ_if_* conforms to a normal distribution of $\text{N }(\xi_{i},\tau_{i}^{2})$, and the parameter $\delta_{if}^{2}$ conforms to an inverse gamma distribution of Inverse Gamma ${\text{Γ}}^{-1}\left(\lambda_{i},\;\theta_{i}\right)$.


$$\varphi_{i}\sim \text{ N }\left(\xi_{i},\;\tau_{i}^{2}\right)\text{ and }\delta_{i}^{2}\sim {\text{ Γ}}^{-1}\left(\lambda_{i},\;\theta_{i}\right)$$

#### 2.1.4 Category-ComBat multicenter effect adjustment

Based on the distribution assumptions described above, the empirical Bayesian parameter estimation method is employed to estimate the posterior distribution parameters $\delta_{if}^{2*}$ and $\varphi_{if}^{*}$ for *Z_ijfc_*.


$$\varphi_{if}^{*}=\frac{\tau_{i}^{2}n_{i}{\hat{\varphi }}_{if}+\delta_{if}^{2^{*}}{\hat{\xi }}_{if}}{n\tau_{i}^{2}+\;\delta_{if}^{2}}\text{ and }\delta_{if}^{2^{*}}=\frac{{\bar{\theta }}_{i}+\frac{1}{2}\sum_{j}{\left(Z_{ijf}-\varphi_{if}^{*}\right)}^{2}}{\frac{n_{i}}{2}+{\bar{\lambda }}_{i}-1}$$

“Category Parameter” ω was introduced as adjustment parameter for scaling the sample data in the final data adjustment formula (Equation 3). The purpose of ω is to enhance the reproducibility of features.

(3) $$Y_{ijfc}^{*}=\frac{\omega {\hat{\sigma }}_{f}}{{\hat{\delta }}_{if}^{*}}\left(Z_{ijfc}-{\hat{\varphi }}_{if}^{*}\right)+{\hat{\alpha }}_{fc}+X{\hat{\beta }}_{f}$$

The “Category Parameter” ω is a coefficient between 0 and 1. It proportionally compresses the distribution of the harmonized data with the center of the category position center *α_fc_*. As a result, it can preserve the reproducibility of the features without compromising their discriminative power.

### 2.2 Datasets

Three publicly available lung CT datasets from The Cancer Imaging Archive (TCIA; https://www.cancerimagingarchive.net/) were included in this study. No ethical concerns apply as all datasets are de-identified and publicly released.

#### 2.2.1 Dataset 1 — LIDC-IDRI

This dataset includes 1,018 patients with benign or malignant lung nodules. All CT images are stored in DICOM format; nodule segmentation data are stored in XML format. Four experienced thoracic radiologists independently annotated each nodule in a blinded fashion, followed by cross-review of one another’s assessments. Malignancy likelihood was rated using a five-level scale: *Highly Unlikely*, *Moderately Unlikely*, *Indeterminate*, *Moderately Suspicious*, and *Highly Suspicious*.

#### 2.2.2 Dataset 2 — NSCLC-radiomics

This dataset comprises 422 patients with pathologically confirmed non-small cell lung cancer (NSCLC); mean age 67.5 years (range 33–91), with 132 females and 290 males. Tumor segmentation was manually delineated by expert radiation oncologists based on fused PET-CT images (lung window: W1700/L−300; mediastinal window: W600/L40; PET window: W30000/L15000) and stored in RTSTRUCT format. Gross tumor volume (GTV) was defined from FDG PET-CT data.

#### 2.2.3 Dataset 3 — lung-PET-CT-Dx

This dataset contains 355 cases of lung cancer (both small cell and non-small cell) confirmed by histology and cytology. Each case includes CT, PET, and fused PET-CT volumes. Lesion segmentation was performed using rectangular bounding boxes by experienced radiologists; one radiologist provided initial annotations, which were subsequently reviewed by four others.

Each dataset was treated as an independent imaging center, yielding a three-center study design.

### 2.3 Data preprocessing

As show in **Figure 2**, A total of 1,789 pulmonary nodule CT cases were initially collected from three publicly available datasets, including LIDC-IDRI (n = 1012), NSCLC-Radiomics (n = 422), and Lung-PET-CT-Dx (n = 355).

**Figure 2:**
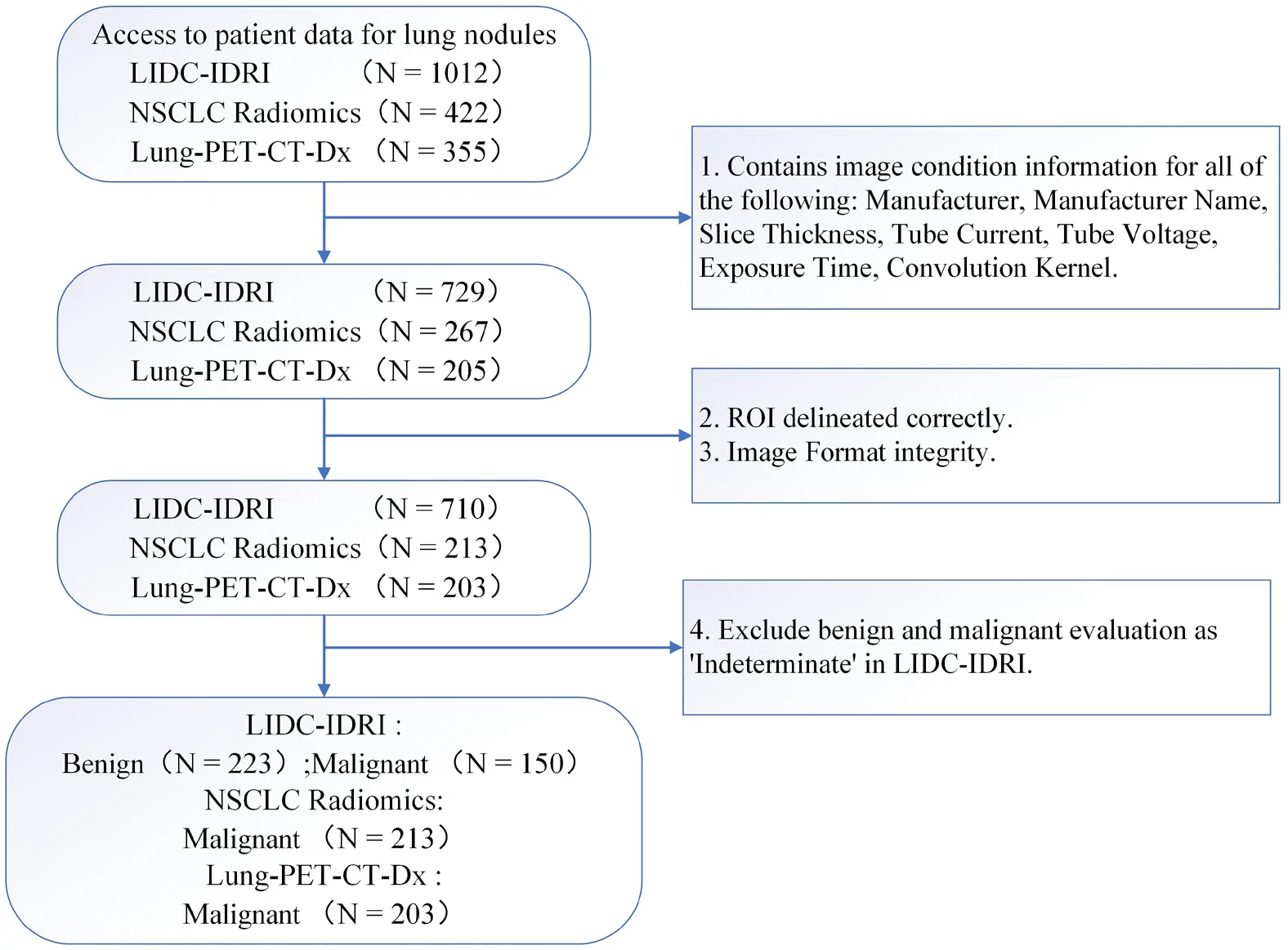
Illustrates the complete inclusion and exclusion process for the study population.

To ensure complete image acquisition information for the proposed Category-ComBat harmonization algorithm, seven acquisition parameters were extracted from the DICOM headers of each case, including manufacturer, manufacturer model, slice thickness, tube current, tube voltage, exposure time, and convolution kernel. Cases with missing information for any of these parameters were excluded, resulting in the removal of 568 cases. Subsequently, 75 cases were excluded because of incomplete or inaccurate ROI delineation or image format inconsistencies.

For the LIDC-IDRI dataset, pulmonary nodules rated as Highly Unlikely or Moderately Unlikely were classified as benign, whereas nodules rated as Moderately Suspicious or Highly Suspicious were classified as malignant. Nodules with an Indeterminate malignancy rating were excluded. For patients presenting with multiple nodules, only one representative nodule was retained to avoid repeated sampling. Consequently, 337 LIDC-IDRI cases were excluded during this step.

As show in **Table 2**, After applying all inclusion and exclusion criteria, a total of 789 CT cases were included in this study, comprising 223 benign and 566 malignant pulmonary nodules. Specifically, the final dataset consisted of 223 benign and 150 malignant cases from LIDC-IDRI, 213 malignant cases from NSCLC-Radiomics, and 203 malignant cases from Lung-PET-CT-Dx.

**Table 2:** Baseline characteristics of the included datasets.

Characteristic	LIDC-IDRI	NSCLC-Radiomics	Lung-PET-CT-Dx
Cases included in this study	373	213	203
Benign	223	0	0
Malignant	150	213	203
Scanner manufacturers	GE Medical Systems, Siemens, Toshiba	Siemens	GE Medical Systems, Philips, Siemens
Slice thickness (mm)	0.6–4.0	N/A*	N/A*
Tube voltage (kVp)	120–140	120–140	80–140
Tube current (mA)	40–581	50–461	100–454

* N/A indicates that the slice thickness information was not available in the corresponding dataset.

As show is **Figure 3**, The radiomics features were extracted from the region of interest using the open-source Python program Pyradiomics 3.0.1. Total of 93 features were extracted. These features were grouped based on their matrix type, including First orders, Glcm (Gray-Level Co-occurrence Matrix), Gldm (Gray-Level Dependence Matrix), Glrlm (Gray-Level Run Length Matrix), Glszm (Gray-Level Size Zone Matrix), and Ngtdm (Neighborhood Gray-Tone Difference Matrix). The extraction process complied with the IBSI Standardization Initiative (25).

**Figure 3:**
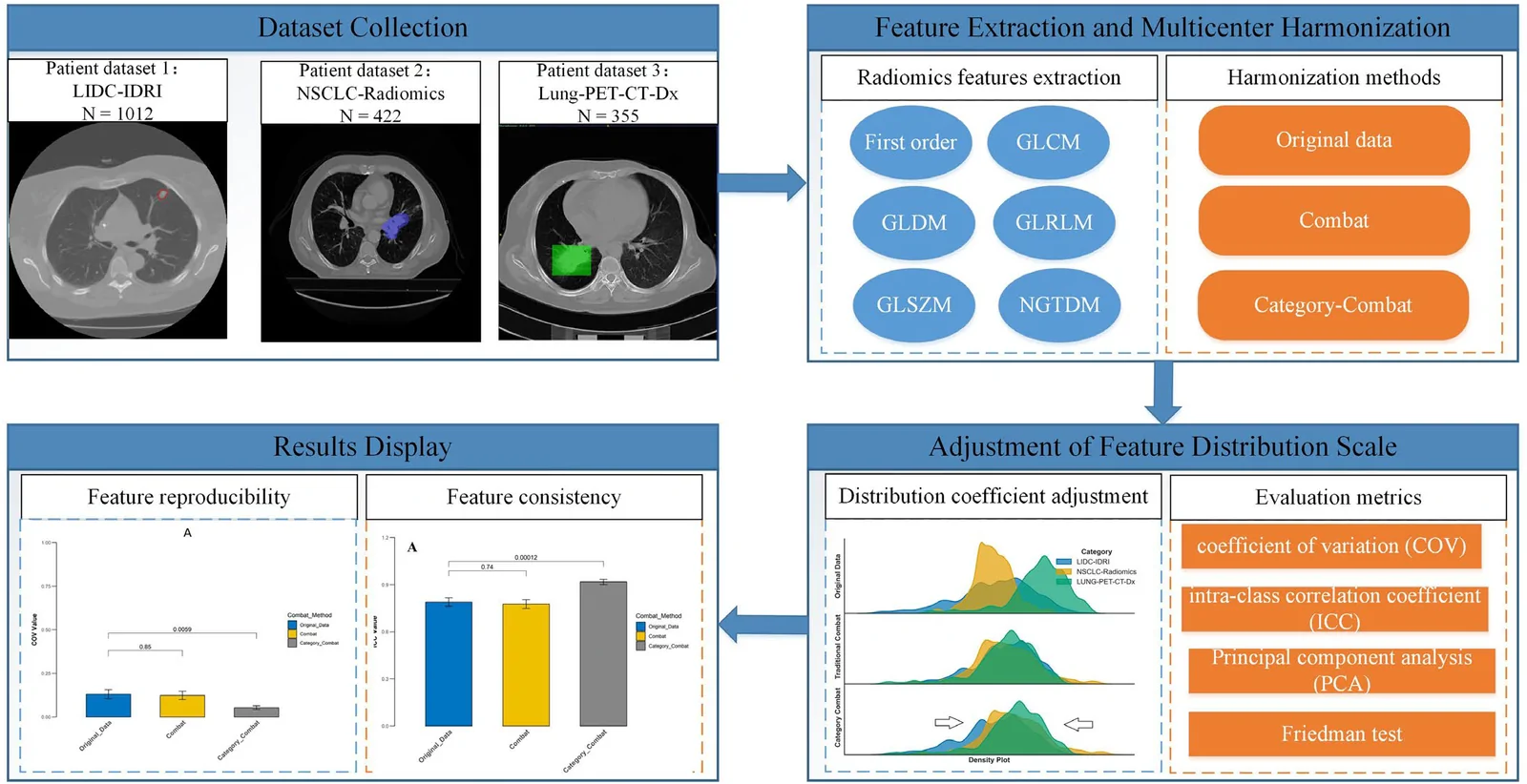
Flowchart of multicenter harmonization algorithm evaluation.

All CT image voxel values were linearly normalized to the range [0, 1]. Isotropic voxel reconstruction was applied with voxel spacing resampled to a uniform 1:1:1 ratio. Gray-level intensities were subsequently discretized into 25 distinct intensity bins. Nodule segmentations were derived directly from each dataset’s provided annotations; all data were converted to NIfTI (NII) format for downstream processing. All shape-related features were excluded from the Lung-PET-CT-Dx dataset, as all ROIs in that dataset are represented as rectangular bounding boxes.

### 2.4 Machine learning model building

The Least Absolute Shrinkage and Selection Operator (LASSO) was utilized to identify the most diagnostically relevant radiomics features. Subsequently, various machine learning models (logistic regression, random forest, support vector machine, and neural networks) were constructed to classify benign and malignant nodules. All implementations were conducted using Python 3.7 and Scikit-Learn 1.0.2.

To strictly prevent data leakage during multicenter harmonization, the dataset was partitioned into a 70% training set and a 30% independent test set prior to any harmonization steps. The Category-ComBat parameters (including the empirical Bayes estimates and category location centers) were fitted exclusively on the training set. The test set was subsequently transformed using these pre-estimated training parameters. For baseline comparisons, models were also trained and evaluated on pre-harmonized (raw) data and traditional ComBat-harmonized data.

Regarding model hyperparameters:

- Logistic Regression: L1 regularization, ‘lbfgs’ optimizer, optimization tolerance of 0.01.
- Random Forest: 100 trees, ‘Gini’ impurity criterion, minimum samples split of 2, maximum depth of 8.
- SVM: RBF kernel, polynomial degree 3, regularization parameter (*C*) of 2, and ‘scale’ gamma.
- Neural Network (MLP): Stochastic Gradient Descent (SGD) solver, L2 regularization, 5 hidden layers with 20 neurons each, learning rate of 0.01, and a maximum of 300 iterations.

### 2.5 Statistical analysis

All statistical analyses were conducted in Python 3.7 and R 4.1.3 (RStudio). The harmonization performance of Category-ComBat was compared with that of the traditional ComBat algorithm. Inter-center reproducibility of radiomic features before and after harmonization was evaluated using the Intraclass Correlation Coefficient (ICC) based on a two-way random-effects model with absolute agreement (ICC(2,1)). This model was selected because both imaging centers and subjects were considered random effects, making it appropriate for assessing reproducibility across multiple centers. Coefficient of Variation (COV) was calculated to evaluate the relative variability of each feature across centers:


$$\text{COV}=\frac{SD}{\bar{X}}\times 100\%$$

where SD is the standard deviation and $\bar{X}$ is the mean across centers.

Principal Component Analysis (PCA) was applied in three dimensions to visually assess the degree of distributional separation and overlap between centers before and after harmonization. Q-Q plots were used to evaluate the goodness-of-fit of the harmonized feature distributions to a theoretical normal distribution. Probability density distributions were plotted to visualize the effect of each ω value (0.8, 0.6, 0.4, 0.2) on the scale of feature distributions, enabling selection of the optimal distribution coefficient. Wilcoxon signed-rank tests were conducted to compare radiomic feature distributions between benign and malignant categories before and after harmonization. To control for multiple comparisons across the 93 radiomic features, P values from the Friedman tests performed after harmonization were adjusted using the Benjamini–Hochberg false discovery rate (FDR) procedure, with an adjusted P< 0.05 considered statistically significant. Model performance was quantified using accuracy, precision, recall, F1 score, and the Area Under the Receiver Operating Characteristic Curve (AUC).

## 3 Results

### 3.1 Friedman test

Friedman’s test results are summarized in **Table 3**. Prior to harmonization, 93 radiomic features exhibited statistically significant between-scanner differences (P< 0.05). Following Category-ComBat harmonization, significant differences were no longer detected in 88 of 93 features (FDR-adjusted P > 0.05), demonstrating that Category-ComBat effectively reduces multicenter effects in radiomic signatures.

**Table 3:** Harmonization test for Friedman’s test between different scanners.

Features names	Without category-combat	With category-combat (FDR P)
Firstorder Minimum	<0.05	0.0008
Glcm Autocorrelation	<0.05	0.0214
Glcm Cluster Prominence	<0.05	0.0187
Glcm Cluster Shade	<0.05	0.0385
Gldm High Gray Level Emphasis	<0.05	0.0442
**Others 88 Radiomics features**	<0.05	>0.05

Note: Bold text indicates the remaining 88 radiomics features not individually listed in the table.

### 3.2 Boxplot analysis

**Figure 4** presents boxplots of representative GLCM features extracted from the LIDC-IDRI dataset acquired using GE Medical Systems and Siemens CT scanners. The original data distributions showed statistically significant between-category differences (P< 0.05). After ComBat harmonization, the position centroids of the two categories were aligned (P > 0.05), but this alignment simultaneously weakened the discriminative power of the features. Category-ComBat, by contrast, harmonized the between-scanner distributional differences while preserving the statistically significant between-category differences (P< 0.05). Additionally, the scatter distributions within boxplots indicate that Category-ComBat drives the feature distributions toward normality.

**Figure 4:**
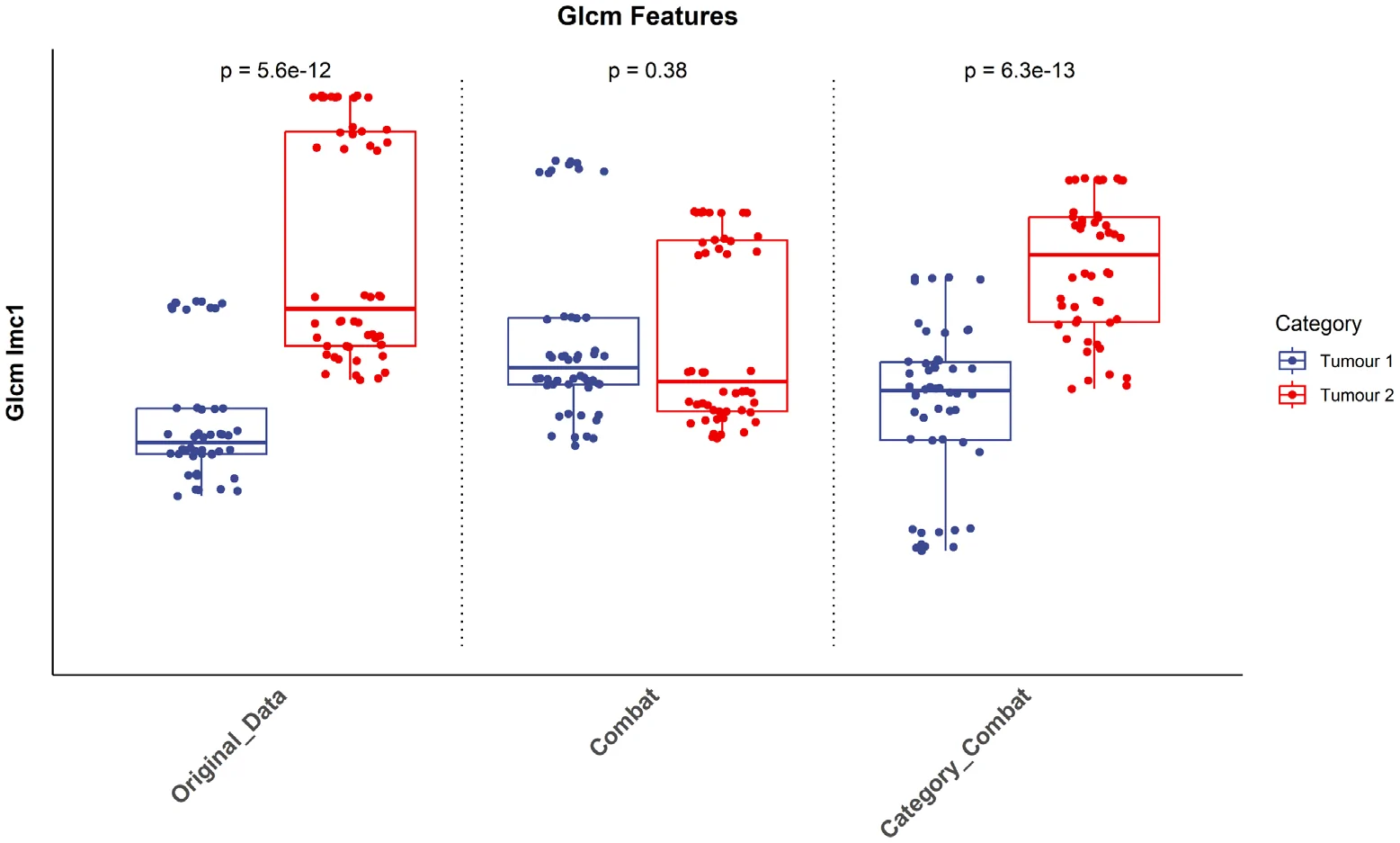
Box plots of the distribution of Glcm Imc1 features extracted from nodules of two categories after harmonization, and the differences were tested using the Wilcoxon.

### 3.3 PCA analysis

**Figure 5** presents PCA results on the three clinical datasets (LIDC-IDRI, NSCLC-Radiomics, and Lung-PET-CT-Dx). In the first row, the original feature distributions show that the three datasets cluster in clearly separated regions with differing dispersions, confirming substantial multicenter effects that preclude direct integration of unharmonized radiomic features.

**Figure 5:**
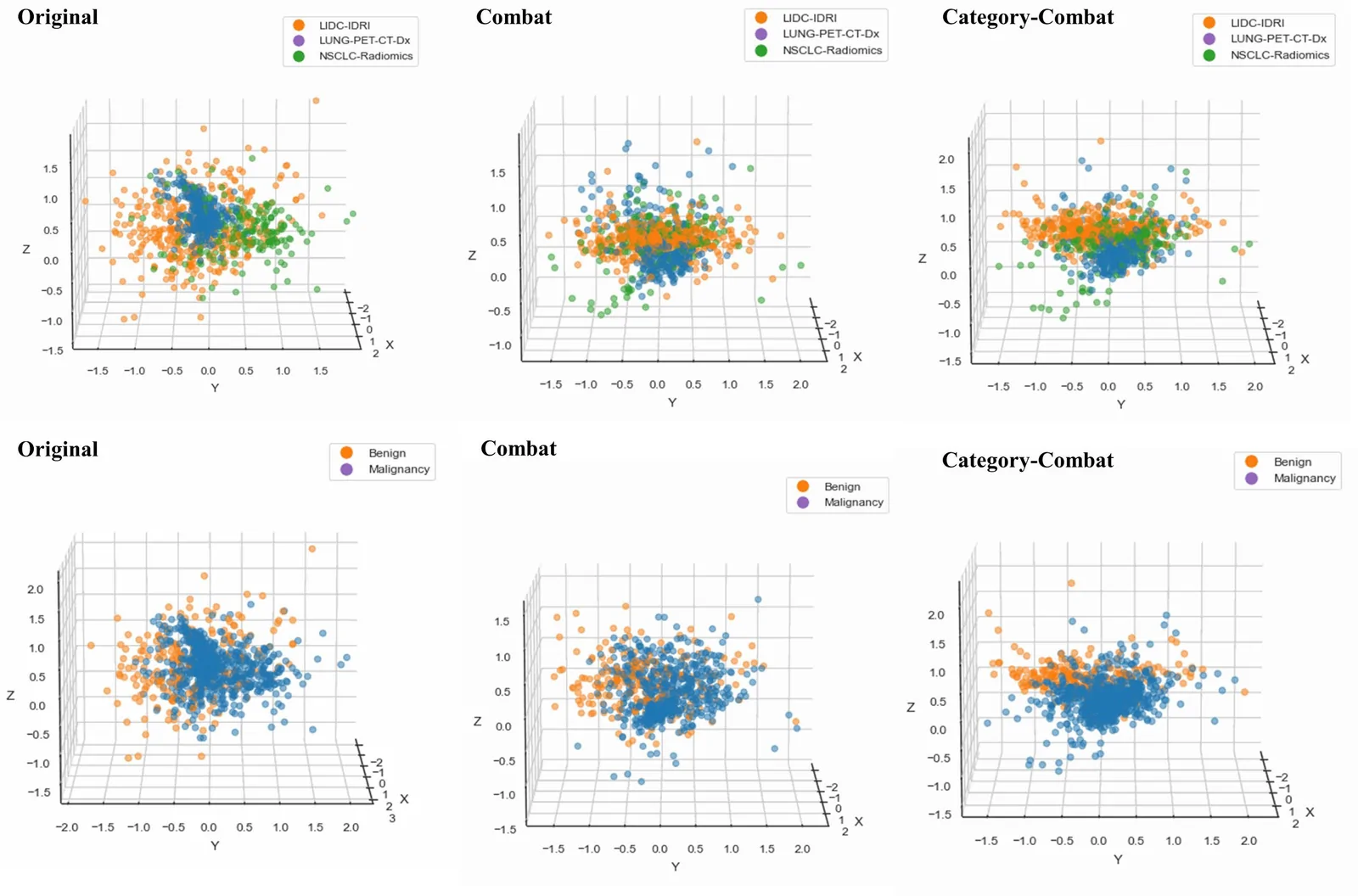
PCA plots of radiomics features of three different datasets and two categories of pulmonary nodules (benign and malignant) harmonized by ComBat and Category-ComBat.

After traditional ComBat harmonization, the cluster positions and dispersions of the three datasets became aligned, confirming ComBat’s harmonization capacity. However, this alignment risks removing clinically meaningful distributional differences between datasets with different patient compositions.

After Category-ComBat harmonization, features from the three datasets were aligned in the second principal component dimension with coordinated dispersion, while differences in cluster positions along the first principal component were preserved. Notably, LIDC-IDRI—which contains both benign and malignant nodules—maintained a distinct distributional cluster position relative to NSCLC-Radiomics and Lung-PET-CT-Dx, which contain exclusively malignant cases. This result demonstrates that Category-ComBat harmonizes technical between-center variability while retaining clinically relevant biological distributional differences.

In the second row, PCA plots stratified by nodule category confirm that the traditional ComBat algorithm erroneously eliminated radiomic feature differences between benign and malignant nodules after harmonization, whereas Category-ComBat preserved robust distributional separation between the two categories across all three datasets.

### 3.4 Coefficient of variation analysis

As show in **Figure 6**, to evaluate the harmonization performance of Category-ComBat in terms of feature consistency, COV was computed across all 93 radiomic features before and after harmonization. As shown in **Figure 6** (left), the mean COV of Original_Data was 0.72, which decreased to 0.58 following traditional ComBat harmonization and further decreased to 0.51 after Category-ComBat harmonization. Statistical comparison revealed no significant difference between Original_Data and ComBat (p = 0.14), whereas the difference between Original_Data and Category-ComBat reached statistical significance (p = 0.00023), indicating that Category-ComBat achieves a more substantial reduction in inter-center feature variability than traditional ComBat.

**Figure 6:**
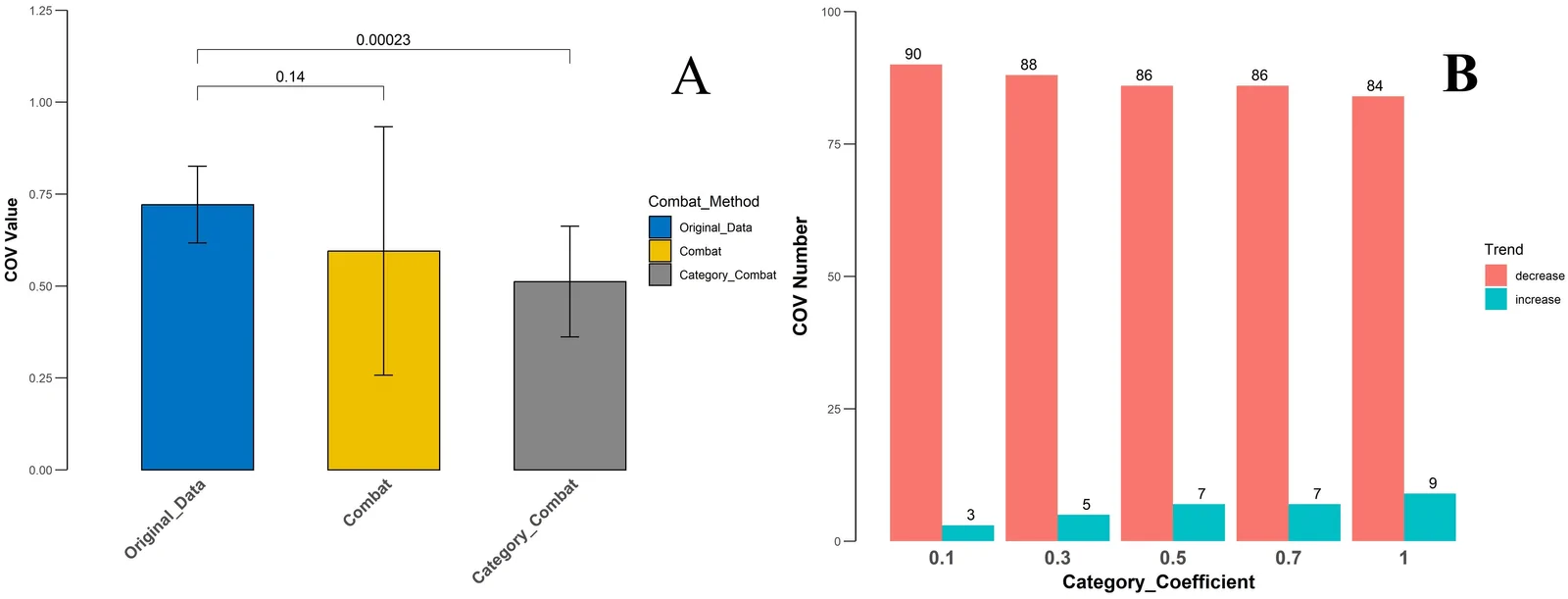
**(A)** the mean coefficient of variation (COV) of 93 radiomics features after applying ComBat and Category-ComBat harmonization methods, and the group differences were tested using the Wilcoxon test. **(B)** The number of radiomics features with COV values in different ranges after harmonization.

To further investigate the effect of the distribution coefficient ω on COV, we analyzed the number of features exhibiting a decreasing or increasing COV trend across five ω values (ω in {0.1, 0.3, 0.5, 0.7, 1.0}). As shown in **Figure 6** (right), the number of features with decreased COV ranged from 84 (ω = 1.0) to 90 (ω = 0.1), while the number of features with increased COV ranged from 3 (ω = 0.1) to 9 (ω = 1.0). These results demonstrate that smaller values of ω yield greater improvements in feature consistency; however, given the risk of over-compression and biological information loss at extremely low ω values, a range of 0.5 ≤ ω ≤ 0.9 is recommended as a practical balance between harmonization effectiveness and the preservation of discriminative feature information. As shown in **Supplementary Figure S2**, the impact of different distribution coefficient (ω) values on feature reproducibility was investigated.

### 3.5 Intraclass correlation coefficient analysis

**Figure 7A** presents the distribution of ICC values across all 93 radiomic features before and after harmonization. Following Category-ComBat harmonization, ICC values increased significantly compared to the original unharmonized data (P< 0.05), indicating that Category-ComBat substantially enhances between-center feature consistency by removing systematic factor variability. The traditional ComBat algorithm, by contrast, produced minimal improvement in ICC values. The effects of different distribution coefficient (ω) values on the mean ICC are shown in **Supplementary Figure S1**.

**Figure 7:**
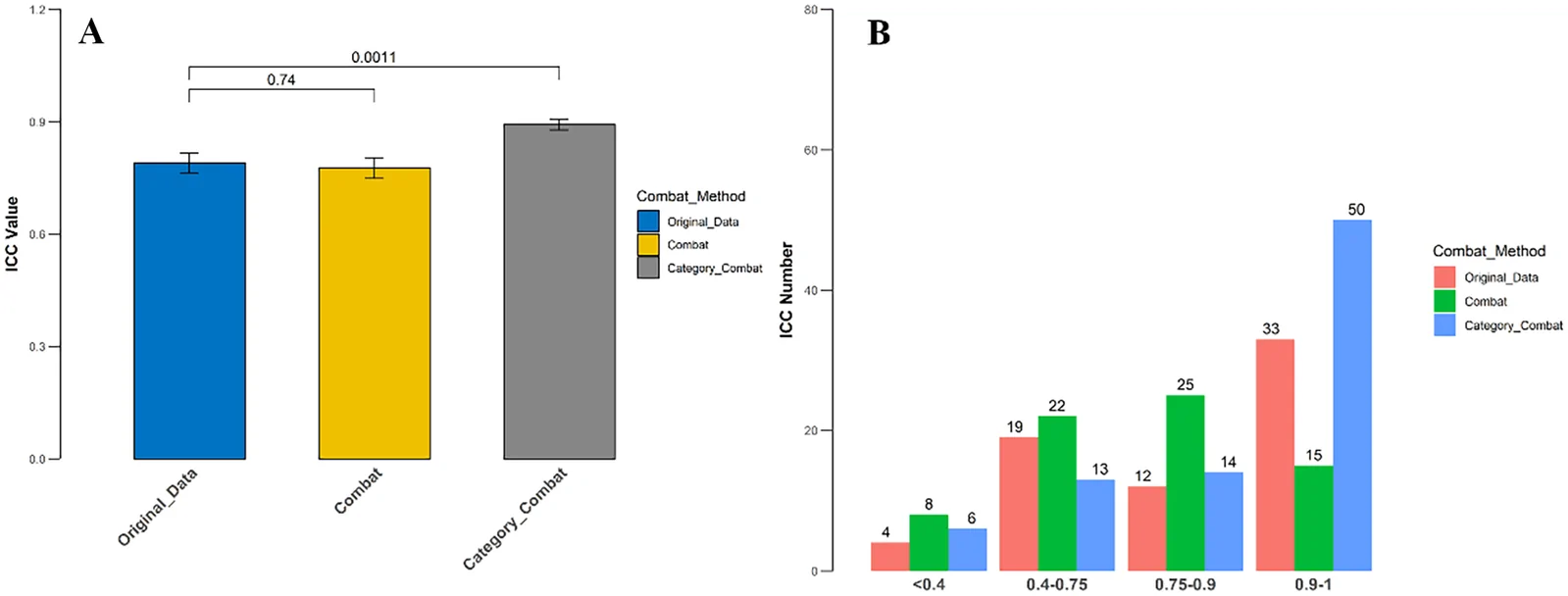
**(A)** Mean intraclass correlation coefficient (ICC(2,1), 95% confidence interval) of 93 radiomic features after ComBat and Category-ComBat harmonization. Group differences were assessed using the Wilcoxon signed-rank test. **(B)** The number of radiomics features with ICC(2,1) values in different ranges after harmonization.

**Figure 7B** categorizes features by ICC range (excellent: 0.90–1.00; good: 0.75–0.90; acceptable: 0.40–0.75; poor: 0.00–0.40). After Category-ComBat harmonization, the number of features with excellent ICC increased from 33 to 50. Conversely, after ComBat harmonization, the number of excellent-ICC features decreased to 15. These results confirm that Category-ComBat’s explicit removal of systematic factor variability yields superior feature consistency relative to traditional ComBat harmonization.

### 3.6 Distribution assumption validation via Q-Q plots

As shown in formula 3, we assume that the standardized data *Z_ijfc_* follows a normal distribution, $Z_{ijfc}\sim N\;\left(\varphi ,\delta^{2}\right)$. The mean *φ* of the standardized data *Z_ijfc_* is assumed to follow a normal distribution, $\varphi_{i}\sim \text{ N }\left(\xi_{i},\tau_{i}^{2}\right)$, and the variance *δ*^2^ is assumed to follow an inverse gamma distribution, $\delta_{i}^{2}\sim {\text{ Γ}}^{-1}\left(\lambda_{i},\theta_{i}\right)$. A Quantile-Quantile (Q-Q) plot is an important tool for assessing the conformity between the actual data distribution and its assumed theoretical distribution. The closer the distribution of actual data distribution is to the theoretical distribution, the more accurate the estimation of the posterior distribution parameters of the model.

As shown in **Figure 8**, the distribution of the standardized data *Z_ijfc_* becomes closer to a normal distribution after removing the systematic factor component. This indicates that by removing components of variability attributed to systematic factors, the assumed distribution for standardizing data in the Category-ComBat algorithm aligns more closely with the actual data distribution. The systematic factor component represents specific factors influencing the consistency of CT image features. The traditional ComBat algorithm is unable to address this bias, resulting in a deficiency in removing the multicenter effects of radiomics features. To more accurately remove the multicenter effect, it is important to remove the affected portion of the systematic factor component.

**Figure 8:**
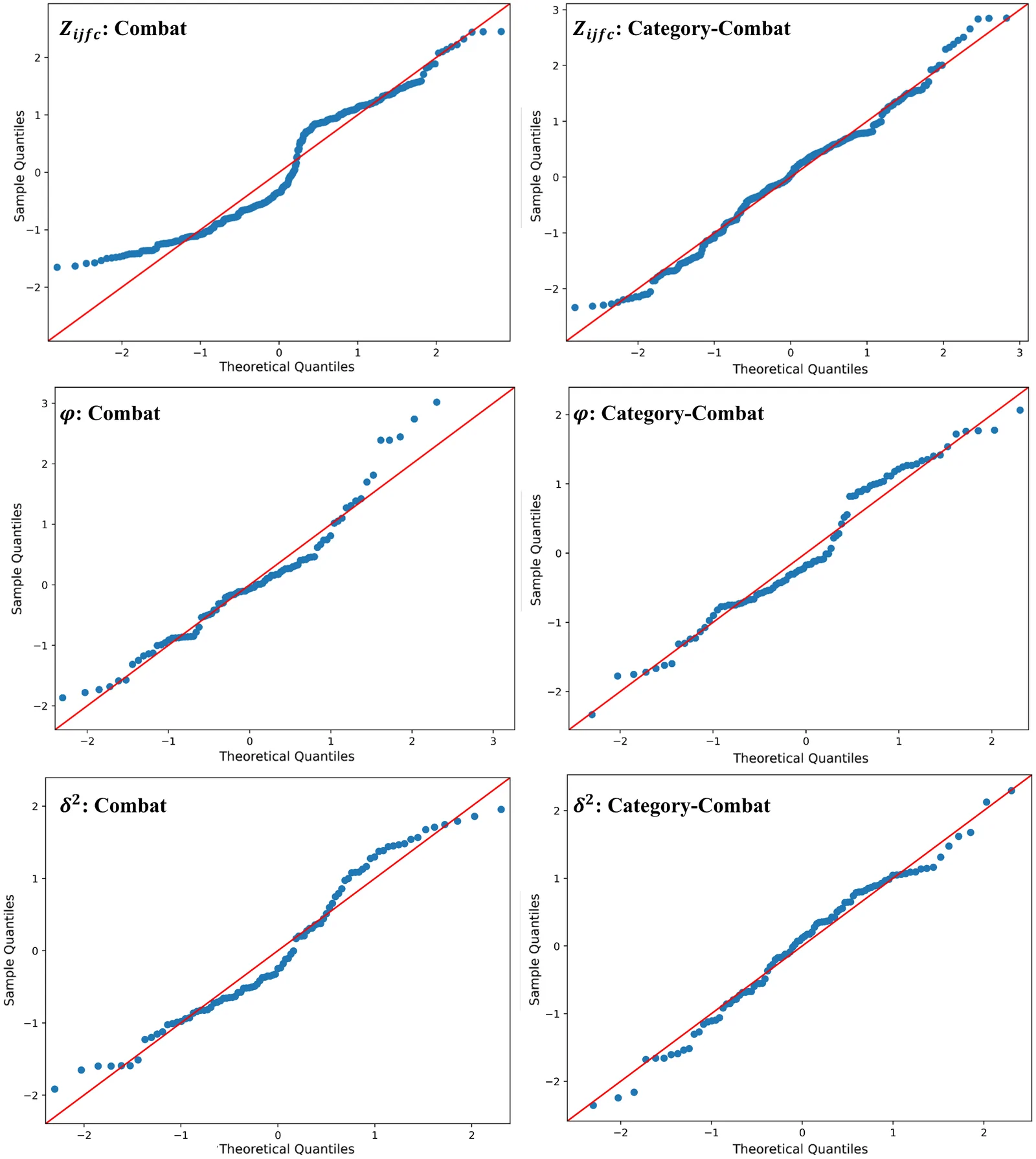
The QQ plot of the normalized data *Z_ijfc_* and *φ* and *δ*^2^ prior distribution after ComBat and Category-ComBat harmonization.

The parameter assumption *φ* represents the mean of the standardized data *Z_ijfc_*, and compared to the traditional ComBat algorithm, the *φ* estimated by the Category-ComBat algorithm is closer to a normal distribution. The parameter *δ*^2^ represents the variance of the standardized data *Z_ijfc_*, and the *δ*^2^ estimated by the Category-ComBat algorithm is closer to an inverse gamma distribution.

Therefore, after removing the systematic factor component, the aforementioned distribution assumptions become more accurate. Through the process of empirical Bayesian parameter estimation, the posterior parameters ${\hat{\varphi }}_{if}^{*}$ and ${\hat{\delta }}_{if}^{2*}$ of the harmonized feature data are more precise. Overall, after removing the distribution of the systematic factor component, the model fits the real data distribution more closely. The Category-ComBat algorithm can better coordinate the multicenter effects in radiomic features caused by complex image conditions.

### 3.7 Machine learning classification evaluation

**Table 4** presents the predictive performance of four distinct machine learning algorithms (Logistic Regression, Random Forest, SVM, and Neural Network) evaluated on the independent 30% hold-out test set. Models were trained and tested using three data configurations: original unharmonized data, traditional ComBat-harmonized data, and Category-ComBat-harmonized data.

**Table 4:** Classification performance of four different machine learning models after ComBat coordination and Category-ComBat coordination.

Model	Coordination method	Accuracy	Precision	Recall	F1	AUC
Logistic regression	Original data	0.88 (0.81-0.94)	0.88 (0.83-0.92)	0.92 (0.88-0.96)	0.92 (0.88-0.96)	0.89 (0.88-0.95)
	ComBat	0.72 (0.64-0.80)	0.76 (0.71-0.82)	0.92 (0.87-0.97)	0.89 (0.81-0.97)	0.75 (0.60-0.91)
	**Category-ComBat**	**0.92** **(0.88-0.96)**	**0.97** **(0.94-0.99)**	**0.93** **(0.87-0.98)**	**0.94** **(0.91-0.97)**	**0.94** **(0.91-0.97)**
Random Forest	Original data	0.86 (0.82-0.91)	0.91 (0.87-0.96)	0.90 (0.84-0.96)	0.90 (0.87-0.93)	0.88 (0.77-0.95)
	ComBat	0.84 (0.78-0.90)	0.89 (0.82-0.96)	0.94 (0.84-0.96)	0.89 (0.85-0.93)	0.86 (0.77-0.94)
	**Category-ComBat**	**0.97** **(0.95-0.99)**	**0.95** **(0.93-0.97)**	**0.98** **(0.96-0.99)**	**0.98** **(0.96-0.99)**	**0.97** **(0.94-0.99)**
SVM	Original data	0.82 (0.87-0.88)	0.85 (0.79-0.90)	0.91 (0.85-0.97)	0.93 (0.89-0.97)	0.84 (0.78-0.95)
	ComBat	0.81 (0.74-0.88)	0.83 (0.78-0.87)	0.94 (0.89-0.99)	0.88 (0.84-0.92)	0.81 (0.70-0.93)
	**Category-ComBat**	**0.97** **(0.94-1.00)**	**0.97** **(0.95-1.00)**	**0.98** **(0.93-1.00)**	**0.98** **(0.90-1.00)**	**0.96** **(0.95-0.97)**
Neural Network	Original data	0.83 (0.79-0.88)	0.84 (0.81-0.88)	0.94 (0.89-0.98)	0.89 (0.86-0.92)	0.85 (0.80-0.93)
	ComBat	0.71 (0.64-0.77)	0.74 (0.70-0.78)	0.81 (0.75-0.87)	0.91 (0.87-0.96)	0.75 (0.53-0.78)
	**Category-ComBat**	**0.84** **(0.79-0.89)**	**0.84** **(0.79-0.89)**	**0.97** **(0.94-1.00)**	**0.90** **(0.87-0.93)**	**0.88** **(0.78-0.97)**

Bold values indicate the best performance.

Notably, models trained on traditional ComBat-harmonized data generally exhibited a decrease in the Area Under the Curve (AUC) and other metrics compared to the original data. This confirms our hypothesis that traditional global harmonization inadvertently removes disease-specific discriminative signals alongside batch effects, thereby degrading classifier performance.

Conversely, the implementation of Category-ComBat substantially enhanced the predictive capacity across all machine learning architectures. By effectively eliminating scanner-induced noise while safeguarding diagnostic-specific feature locations, Category-ComBat provided the models with high-fidelity, generalizable signals. The Random Forest classifier achieved the most superior performance, yielding an AUC of 0.97 (95% CI: 0.94-0.99), an Accuracy of 0.97 (95% CI: 0.95-0.99), and an F1-score of 0.98 (95% CI: 0.96-0.99) on the independent test set.

The ROC curves **Figure 9** further corroborate these findings, visibly demonstrating that Category-ComBat systematically out-performs both the unharmonized and traditional ComBat approaches. These results definitively validate the superiority of the Category-ComBat algorithm in robustly resolving multicenter batch effects while maximizing the clinical utility of radiomic biomarkers.

**Figure 9:**
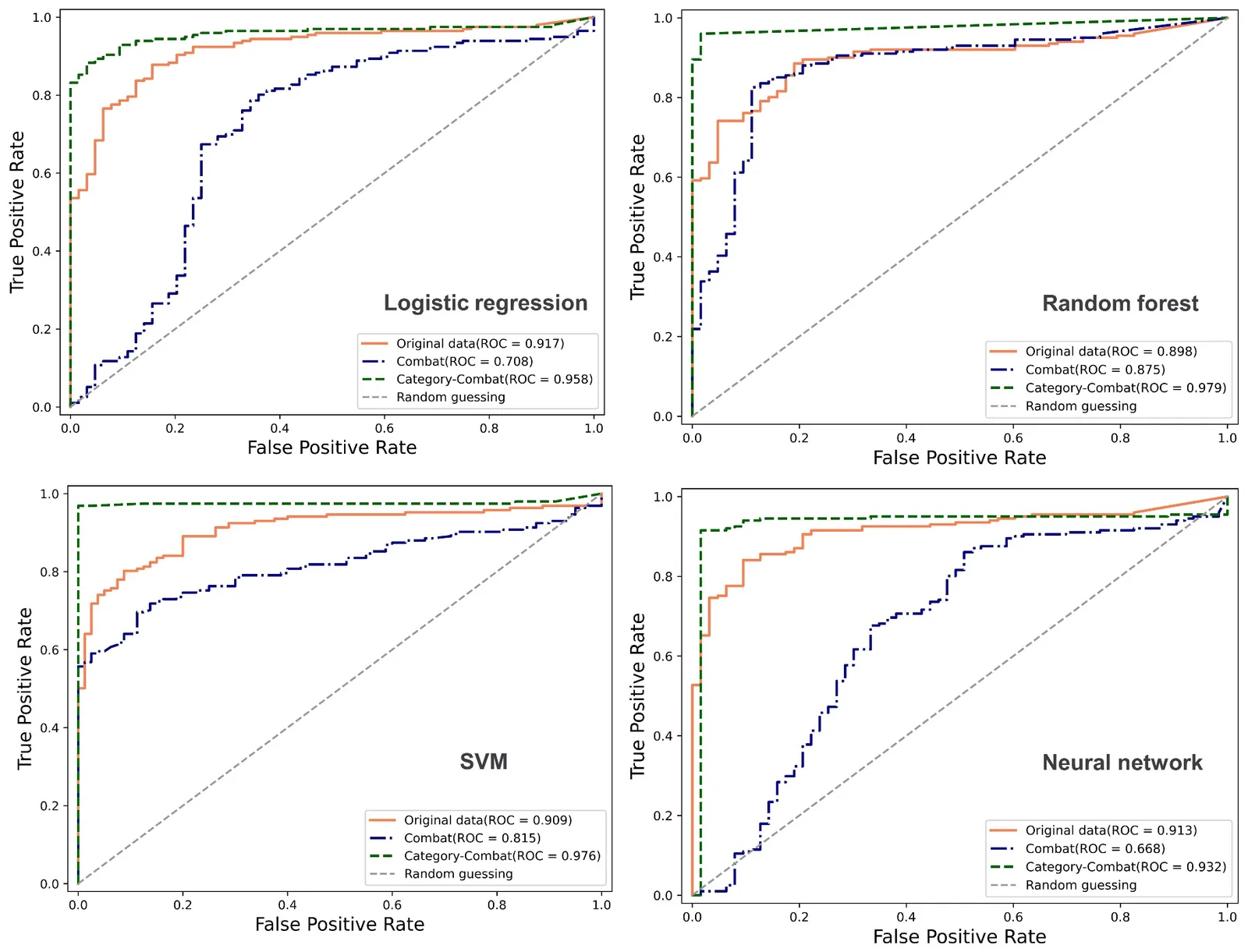
ROC curves of four different machine learning models after ComBat coordination and Category-ComBat coordination.

## 4 Discussion

The reproducibility, discriminative power, and cross-center consistency of radiomic features are critical determinants of the robustness and generalizability of radiomics models. Recent studies have also emphasized that improving radiomic feature robustness is essential for reliable multicenter radiomics analysis, as imaging and reconstruction variability can substantially influence feature stability and downstream model performance (27). Recent studies have demonstrated that harmonization methods, particularly ComBat, can substantially improve the robustness of multicenter radiomic analysis and enhance downstream machine learning performance. Our proposed Category-ComBat further extends this concept by incorporating biological category information into the harmonization process, aiming to preserve discriminative biological variability while reducing technical inter-center effects (28).

Category-ComBat algorithm extends the traditional ComBat algorithm by introducing two key innovations: (1) the categorization of imaging condition factors into systematic and random components, enabling personalized and targeted correction of their respective effects on radiomic features; and (2) a feature distribution coefficient (ω) that adjusts the scale of feature distributions to further improve reproducibility and consistency while preserving discriminative information.

The traditional ComBat algorithm was originally developed to address batch effects in genomic data (15). In the field of medical imaging, however, the sources of inter-center variability are considerably more complex, encompassing acquisition parameters (scanner model, tube voltage, tube current, scanning protocol), reconstruction parameters (reconstruction kernel, reconstruction field of view), and environmental factors (vibration, motion artifacts, operator variation) (29). These heterogeneous imaging conditions represent a major obstacle to the effective multicenter harmonization of radiomic features. In contrast to traditional ComBat algorithms, including traditional ComBat, B-ComBat algorithm (30), M-ComBat algorithm (31), ComBat-seq algorithm (32) and other ComBat-derived algorithm (33) — Category-ComBat explicitly partitions these sources of variability. Acquisition and reconstruction parameters that exert systematic, reproducible effects on features are classified as systematic factors, while environmental disturbances and operator-related variability that introduce stochastic variation are classified as random factors.

To mitigate these adverse effects, Category-ComBat introduces a systematic factor matrix *X_r_*, which captures the personalized imaging conditions for each case during feature extraction. By estimating the regression vector $\hat{\beta }r$ via the matrix ***X****_r_*, the algorithm removes the systematic factor component *X_r_β_r_* in a case-specific manner, enabling more accurate elimination of deterministic inter-center bias. For random factors, additive and multiplicative error terms $\gamma_{if}+\delta_{if}\epsilon_{ijfc}$ model their stochastic effects, an approach that has been demonstrated to effectively capture random-factor-induced variation (15). This integrated design robustly addresses the full spectrum of imaging condition variability, improving both the modeling accuracy of the algorithm and the consistency and robustness of the harmonized features.

We collected a total of 1789 lung nodule cases from three publicly available datasets — LIDC-IDRI, NSCLC-Radiomics, and Lung-PET-CT-Dx — encompassing both benign and malignant nodules across three imaging centers. Three-dimensional PCA visualization confirmed that multicenter effects in radiomic features were effectively removed by Category-ComBat while discriminative differences between categories were preserved, a property not consistently achieved by the traditional ComBat algorithm, which often compromises feature discriminability during harmonization (34). Intraclass correlation coefficient (ICC) analysis further demonstrated that after categorizing and removing the systematic factor component, Category-ComBat significantly reduced inter-center variability in radiomic features, enhancing both their consistency and robustness.

This study used the reference segmentations provided by the original public datasets and did not perform additional manual delineation. Therefore, inter- and intra-observer segmentation reproducibility was not evaluated, which should be considered a limitation of the present study. Future work using repeated manual segmentations will allow assessment of the combined effects of segmentation variability and feature harmonization.

Similar to the approach adopted by Johnson et al. (15). to evaluate the fit of the traditional ComBat model using Q-Q plots, we employed Q-Q plots to assess the influence of the systematic factor component ***X_r_β_r_*** on the distributional assumptions underlying Category-ComBat. The results demonstrated that the empirical distribution of the standardized data *Z_ijfc_* aligned substantially more closely with the assumed normal distribution $N\left(\text{φ},{\text{δ}}^{2}\right)$ after removing the systematic factor component. Correspondingly, the prior mean parameter *φ* exhibited better agreement with its assumed normal prior, and the prior variance parameter *δ*^2^ more closely conformed to its assumed inverse gamma prior. These improvements validate the distributional assumptions of the model and provide a more accurate basis for empirical Bayesian parameter estimation, ultimately yielding more precise posterior parameters ${\hat{\varphi }}_{if}^{*}$ and ${\hat{\delta }}_{if}^{2*}$ and enhancing the overall harmonization precision of the algorithm.

To further characterize the role of the systematic factor component, we performed Wilcoxon tests on the *X_r_β_r_* values of 93 radiomic features and analyzed the resulting p-value distributions between benign and malignant lung nodule categories. A p-value distribution skewed toward zero indicates that the apparent discriminative ability of certain features is not intrinsic to the biological signal, but rather a spurious consequence of systematic imaging variability. Such detrimental variation undermines feature robustness and leads to features that appear effective within a single center but fail to generalize across institutions — a critical limitation that Category-ComBat is specifically designed to address.

Beyond systematic factor removal, this study introduces the distribution coefficient ω as an additional tuning parameter to further optimize harmonization performance. The coefficient ω adjusts the scale of feature distributions after harmonization: when ω = 1, the feature distribution range is preserved without compression; as ω decreases below 1, the feature distributions are proportionally compressed. Analysis of coefficient of variation (COV) and ICC values across 93 features demonstrated that Category-ComBat at ω = 1 already slightly outperforms traditional ComBat, and that further reductions in ω progressively enhance feature reproducibility and consistency. Nevertheless, ω functions as a double-edged sword: excessively low values compress feature distributions to a degree that may destroy biologically meaningful variation, impairing the discriminative capacity of the radiomic signature and potentially degrading the classification performance of downstream radiomics models. Based on the experimental results of the datasets evaluated in this study, a weighting coefficient in the range of 0.5 ≤ ω ≤ 0.9 provided a favorable balance between harmonization and preservation of biological variability. This recommendation is empirical and may depend on dataset characteristics such as scanner heterogeneity, sample composition, and imaging protocols. Further validation on additional multicenter datasets is warranted before generalizing this recommendation.

Unlike PCA visualization and Wilcoxon analysis, downstream machine learning performance provides direct evidence of discriminative information preservation. The consistently improved AUC observed after Category-ComBat further supports that the proposed harmonization preserves biologically relevant information while reducing technical variability.

Category-ComBat has the potential to facilitate the integration of medical images from different institutions into a harmonized, consistent multicenter radiomic feature repository, providing researchers with more robust disease biomarkers and a more reliable foundation for clinical radiomics applications. However, several limitations should be acknowledged. First, the categorization of imaging condition factors into systematic and random components warrants more detailed and comprehensive validation across diverse imaging protocols and scanner types. Second, additional independent datasets and experimental conditions are needed to further validate the generalizability and effectiveness of the algorithm. Third, the impact of Category-ComBat harmonization on downstream machine learning model performance — including classification accuracy, AUC, and feature selection stability — requires systematic investigation.

Although the present study employed three publicly available datasets to maximize imaging diversity, these datasets differ in disease composition, lesion annotation strategy, segmentation methodology, and imaging workflow. Consequently, biological heterogeneity cannot be completely separated from technical imaging variability. Therefore, the current study should be interpreted as a proof-of-concept evaluation of Category-ComBat under heterogeneous real-world radiomics datasets rather than a definitive validation using clinically matched multicenter cohorts. Future studies will evaluate the proposed algorithm on prospectively collected multicenter datasets with identical clinical tasks, standardized segmentation protocols, and harmonized acquisition criteria to further isolate scanner-related variability.

Despite these limitations, Category-ComBat demonstrates clear advantages in improving the consistency and robustness of multicenter radiomic features, and future work aimed at addressing these limitations will further enhance its practical reliability and clinical applicability.

## 5 Conclusions

In this study, we proposed Category-ComBat, a multicenter radiomic feature harmonization algorithm that extends traditional ComBat by explicitly decomposing imaging condition factors into systematic and random components. By introducing the factor matrix *X_r_* to model case-specific systematic effects, the algorithm enables targeted removal of the systematic factor component $X_{r}\hat{\beta_{r}}$, improving the conformity of standardized data *Z_ijfc_* to the distributional assumptions of the model and yielding more precise empirical Bayesian posterior parameter estimates ${\hat{\varphi }}_{if}^{*}$ and ${\hat{\delta }}_{if}^{2*}$. Furthermore, the introduction of the distribution coefficient ω provides a tunable mechanism to enhance feature reproducibility and consistency, with a recommended range of 0.5 ≤ω≤0.9 for multicenter pulmonary nodule radiomics studies. Experimental results demonstrated that Category-ComBat effectively reduced inter-center variability while preserving discriminative information, outperforming traditional ComBat in terms of ICC and COV across 93 radiomic features. These findings suggest that Category-ComBat offers a robust and flexible solution for multicenter radiomic harmonization, with broad potential for application in large-scale clinical radiomics research. Furthermore, downstream machine learning evaluation demonstrated that Category-ComBat maintained or improved benign–malignant classification performance after harmonization, providing direct evidence that the proposed framework preserves biologically meaningful discriminative information while reducing technical imaging variability.

## Data Availability

Publicly available datasets were analyzed in this study. This data can be found here: cancerimagingarchive.net.
