# Supplementary material for: Category-ComBat with distribution coefficient: a scale-adaptive harmonization algorithm for multicenter radiomic feature standardization
Source: Front Oncol. 2026 Sep 14;16:1896994. doi: 10.3389/fonc.2026.1896994 (PMC13617529; doi:10.3389/fonc.2026.1896994)
Supplement: Supplementary file 1 [file SupplementaryFile1.docx]

As illustrated in Figure S1, we conducted a statistical analysis on the impact of the distribution coefficient "𝜔" on the change in overall ICC for 93 features. The results indicated a noticeable improvement in feature consistency as the distribution coefficient "𝜔" decreased. When the distribution coefficient “𝜔” is set to 0.8. The feature consistency after coordination by the Category-ComBat algorithm is slightly higher than that after coordination by the ComBat algorithm. However, there was no significant difference in the consistency of features harmonized by both algorithms compared to the original data. When the distribution coefficient "𝜔" was set to 0.6. The consistency of features harmonized by the Category-ComBat algorithm was significantly higher than that of features harmonized by the ComBat algorithm. It was notably higher than the consistency of the original data.

**Figure S1: The impact of different "Distribution Coefficients" on the mean intra-class correlation coefficient (ICC) of 93 radiomics features after applying ComBat and Category-ComBat harmonization methods.**

As shown in Figure S2, the impact of different distribution coefficient “𝜔” values on feature reproducibility was investigated. We set the coefficients of distribution coefficient “𝜔” to 0.2, 0.4, 0.6, and 0.8, respectively. The Category-Combat algorithm was used to harmonize the COV values of the radiomics features under each setting. The mean and error of the COV values for 93 radiomic features were then statistically analyzed.

When the distribution coefficient “𝜔” was set to 0.8, both Combat and Category-Combat algorithms showed no significant difference in the reproducibility of radiomic features compared to the original data. However, with a distribution coefficient “𝜔” of 0.6, the reproducibility of radiomic features harmonized by the Category-Combat algorithm significantly improved. As the distribution coefficient “𝜔” decreased, the distribution of features was compressed. It results in a larger improvement in feature reproducibility.

Figure S2: The impact of different distribution coefficient “𝜔” on the mean coefficient of variation (COV) of 93 radiomics features after applying Combat and Category-Combat harmonization methods.
